# Identifying prognostic factors for conservative treatment outcomes in servicemen with chronic exertional compartment syndrome treated at a rehabilitation center

**DOI:** 10.1186/s40779-017-0145-2

**Published:** 2017-11-28

**Authors:** Mariëtte Z. Meulekamp, Peter van der Wurff, Alfred van der Meer, Cees Lucas

**Affiliations:** 1 Research and Development, Military Rehabilitation Centre, Aardenburg, Doorn the Netherlands; 2 Department of Physiotherapy, HU University of Applied Sciences, Utrecht, the Netherlands; 3 Department of Clinical Epidemiology, Biostatistics and Bioinformatics, Academic Medical Centre, University of Amsterdam, Amsterdam, the Netherlands

**Keywords:** Lower leg pain, Observational study, Rehabilitation

## Abstract

**Background:**

Chronic exertional compartment syndrome (CECS) is a condition of pain induced by exercise, and it is characterized by muscle swelling and impaired muscle function in the lower leg. Given the diversity in the diagnosis and treatment of CECS, it is desirable to determine variables pertaining to prognosis and recovery. The purpose of this study is to identify prognostic factors for conservative treatment outcomes in servicemen with CECS who were treated at a Military Rehabilitation Center.

**Methods:**

Patients from all military services were referred from the special unit for lower leg pain at the Central Military Hospital, Utrecht, the Netherlands. Descriptive analysis was used to report the characteristics of the participants and their baseline measurements. Group differences were analyzed using a Student’s *t*-test or Mann-Whitney *U* test, according to the normality of the data distribution. Differences between the pre- and post-intervention outcomes were evaluated using the Wilcoxon signed rank test. To evaluate the magnitude of prognostic factors, a univariate logistic regression analysis was performed. The prognostic factors included age, body mass index, body fat percentage, self-efficacy beliefs, foot malalignment, intramuscular pressure, other comorbidities, protein and creatine use, smoking, alcohol use, complaint duration, physical demands, and duration of military service.

**Results:**

After the rehabilitation period, we observed 25 patients with a successful outcome, which was defined as a reduction in pain (≥ 2 points) during the capacity test measured using a verbal rating scale and 20 patients with an unsuccessful outcome. Factors demonstrating a limited increased odds ratio for an unsuccessful outcome included smoking, alcohol use, intramuscular pressure, a complaint duration of more than 6 months, and physical demands of service. However, these factors did not reach significance.

**Conclusion:**

This study did not identify any prognostic factors that predict the outcome of a rehabilitation program for CECS. A larger sample using an identical design might provide further evidence regarding prognostic factors, which would facilitate development of a model that predicts the outcomes of a rehabilitation program for CECS.

## Background

Chronic exertional compartment syndrome (CECS) is an exercise-related condition of the lower leg that is common in athletes and servicemen. In a recent study performed in the United States, 4100 servicemen with CECS were identified over a 6-year period, with an incidence of approximately 0.49 per 1000 person-years [1]. The incidence of CECS in the military in the Netherlands and other European countries remains unknown.

CECS is a condition of pain induced by exertion and is characterized by tightness, muscle swelling, and pain that is classically localized to the anterolateral area of the lower leg [2]. The pathophysiology of CECS is likely multifactorial in nature, and it may be a product of the fixed volume capacity of muscle compartments, normal or abnormal muscle swelling from activity, abnormally thickened fascia, normal muscle hypertrophy in response to resistance training, or dynamic contraction patterns during gait [3].

Intramuscular pressure (IMP) measurements remain the gold standard for the diagnosis of CECS. However, IMP thresholds vary considerably between studies [4–11].

Studies of CECS treatment have principally focused on the outcomes of surgical management of CECS [12]. Better outcomes have been reported for civilians, with only half of all military service members reporting complete resolution of symptoms after treatment, and 25% or more were unable to return to full duty due to persisting symptoms [13].

Given the rates of CECS in the military and the burden of both surgical management and the management of complications, CECS constitutes a significant liability for military healthcare.

Only a few studies, which are mostly case series, have reported on the success rate of conservative treatment, e.g., massage, gait training, and chemodenervation, for CECS [14–20].

Although prognostic factors for the outcomes of surgical treatment of CECS have been reported, these factors remain controversial in the literature [2, 7, 21, 22]. Similarly, prognostic factors for conservative treatment have not yet been clarified.

To this end, we merged previously examined conservative treatment methods into a multidisciplinary program that combined physical therapy, physical fitness, mental coaching, and application of insoles [23]. Given the general opinion about the multifactorial nature of CECS and the uncertainty regarding diagnosis, we designed a study to examine prognostic factors for the outcomes of a conservative treatment approach for CECS in servicemen treated at a military rehabilitation center.

## Methods

### Patients

This was a prospective study with the aim of identifying prognostic factors for the outcome of a rehabilitation program for CECS in servicemen treated at the rehabilitation center. All patients were referred from the special unit for lower leg pain at the Central Military Hospital, Utrecht, the Netherlands. The staff of this unit included a surgeon, a physiatrist, and a sports physician who were responsible for deciding whether patients met the inclusion criteria for this study.

Patients from all military services of the Netherlands Armed Forces (NAF) between 17 and 45 years old were eligible for this study. Patients referred to the special unit for lower leg pain were screened using the following inclusion criteria: 1) positive IMP measurement (> 30 mmHg) in at least one of the compartments of the lower leg; 2) symptoms of CECS that persisted for >3 months; and 3) CECS as the primary complaint. Patients with other conditions hampering the treatment of CECS as well as patients who had been treated by fasciotomy in the past year or who were not willing to participate in the study were excluded [24].

Ethical approval of this study was obtained by a waiver from the Central Committee for Human Research (CCMO) of the Netherlands and Defense Health Organization (DGO), the Netherlands. All patients provided informed consent.

### Prognostic indicators

All baseline demographic values and prognostic factors were assessed by a trained independent observer at the start of the rehabilitation period at the Military Rehabilitation Centre in Aardenburg. During the initial evaluation, each patient completed a standardized questionnaire and underwent a standardized physical examination. The prognostic value of the following factors was evaluated: duration of military service, age, body mass index (BMI), body fat percentage, self-efficacy beliefs, foot malalignment, IMP measurements, comorbidities, alcohol use, supplement use, smoking status, complaint duration, and the demands of service. BMI was analyzed in a dichotomous manner with a cut-off set at <25 kg/m^2^ (normal weight) and ≥25 kg/m^2^ (overweight) [25]. Body fat percentage was also analyzed dichotomously with a cut-off for low and high body fat percentage set between 15% and 20%, according to age, as defined by Durnin and Wormersley [26]. Self-efficacy beliefs are an important psychosocial determinant of pain behavior and management, and they were measured using the Pain Self-Efficacy Questionnaire (PSEQ) [27]. Foot malalignment was measured using the Foot Posture Index (FPI) with 5 categories analyzed in a dichotomous manner as either ‘normal’ or ‘abnormal’ [28]. IMP measurements were taken from the Central Military Hospital, with the highest score of all measured compartments used in the analysis. The presence of other comorbidities and the use of supplemental protein, creatine, smoking, and alcohol were all analyzed dichotomously as present or absent. The complaint duration was also analyzed dichotomously using a cut-off of 7 months. The level of physical demand was measured on an ordinal scale classifying the physical demands of service into 6 levels, as defined by the NAF. For analysis, the physical demand score was dichotomized as the patient experiencing either low (≤ 3) or high (> 3) physical strain. Finally, the duration of military service was measured in months and analyzed continuously.

The primary outcome measurement at 6 weeks was ‘successful’ or ‘unsuccessful’ rehabilitation, with restoration of function and resolution of pain being the primary goals of rehabilitation. A functional capacity test, which consisted of a 12 min walk or run, was performed at baseline and at 6 weeks. The test was performed by the same observer, who was not involved in the treatment procedure, at the two time points. A ‘successful’ outcome of the rehabilitation program was defined as a reduction in pain (≥ 2 points) during the capacity test, which was measured using the verbal rating scale (VRS) [29]. The VRS is a 1-dimensional pain intensity scale that is administered verbally with pain scored on an 11-point ordinal scale, that ranges from 0 ‘no pain’ to 10 ‘worst pain’. Two secondary outcome measures were evaluated using the numeric rating scale (NRS) and patient specific functional scale (PSFS), both of which were collected at baseline and at the end of the 6-week rehabilitation program at 6 weeks. The NRS is the written equivalent of the VRS [30]. The NRS records the current pain level and the maximum pain over the past week. The PSFS was used to provide insight regarding the capacity for daily activities in patients with CECS, and it was measured on a visual scale ranging from 0 mm (unable to perform activity) to 100 mm (able to perform the activity at pre-morbidity level) [31]. The patient can choose from preselected PFPS items: prolonged standing, walking, running, driving a car, marching, and climbing stairs. Patients were permitted to score a maximum of 3 activities. The overall score of these activities was summated and averaged to obtain a total score out of 100.

### Rehabilitation protocol

All patients followed the rehabilitation protocol in group sessions 3 times a week for 6 weeks at the rehabilitation center. The rehabilitation protocol was divided into an observation stage of 1 week and a treatment stage of 5 weeks. The indication and treatment objectives were defined based on the observation period. Two physical therapists and a sports trainer were involved in all of the group sessions. The main goal of the treatment protocol is to reduce the complaints as much as possible and to balance the load-carrying capacity to increase the employability of the subject. The treatment protocol is divided into 4 modules. First, the “pre-conditions” module is characterized by normalization of joint mobility and restoration of normal muscle tone. Second, the “body awareness physical training” module consists of strength improvement and endurance of muscle power, especially the calf muscles, through walking instructions and stability training of the core and lower extremities. Third, the “posture and movement” module is focused on functional gait training, deviations when standing, adaptation of footwear and/or inlays and core stability training. Fourth, the “behavioral” module involves a graded activity approach. This protocol has previously been described in detail by Meulekamp et al. [23].

When a successful outcome of the rehabilitation program was obtained, patients returned to their unit with tailored advice for further treatment and support to obtain complete recovery and full return to work. In cases of an unsuccessful outcome, the patient was referred for further evaluation to the lower leg pain unit of the Central Military Hospital, Utrecht, the Netherlands.

### Statistics

All data analyses were performed with SPSS version 22 (SPSS Inc., Chicago, IL, USA). Descriptive analysis was used to report the characteristics of the participants and baseline measurements, and the data were tested with the Chi-square test unless otherwise stated. Group differences were analyzed using the Student’s *t*-test or Mann-Whitney *U* test according to the normality of data distribution. Continuous variables are presented as the mean ± SD, unless otherwise noted.

For evaluation of the supplementary patient-reported outcome measurements, we calculated group means with the standard deviation for the differences between baseline and follow-up measurements after 6 weeks. Differences between the pre- and post- intervention outcomes were evaluated using the Wilcoxon signed rank test. To evaluate the magnitude of prognostic factors, a univariate logistic regression analysis was performed, and estimated odds ratios and 95% confidence intervals (CI) were derived.

## Results

**Fig. 1:**
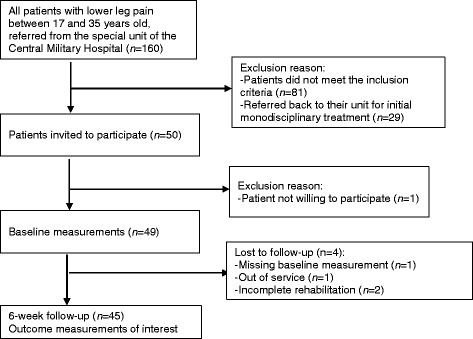
Flowchart of the study population

A total of 45 patients participated in this study. The study flowchart is presented in Fig. 1. The baseline characteristics of the study group are presented in Table 1. No significant differences were found between the groups with respect to continuous data at baseline. Data pertaining to body fat percentage were incomplete; however, this omission (6.7%) is acceptable, and no imputation was required.

**Table 1:** Demographic parameters at baseline between subjects with successful and unsuccessful outcomes

Item	Successful (*n* = 25)	Unsuccessful (*n* = 20)	*P* value	Statistical method
Age (year, median (range))	23.0 (19–40)	22.5 (18–34)	0.96	M-U
Male (*n*)	24	20	0.37	χ^2^
Military service (*n*)			0.23	χ^2^
Army	21	16		
Air force	2	1		
Navy	1	2		
Military police	1	1		
Sports behavior (*n*)			0.26	χ^2^
Military sports	15	9		
Running	14	8		
Soccer	5	5		
Fitness	10	13		
Remaining	2	6		
Duration of military service (month, median (IQR))	21 (12, 55)	32 (10, 82)	0.79	M-U
IMP (mmHg, *x* ± SD)	63.33 ± 15.49	65.01 ± 16.37	0.42	T
BMI (*n*)			0.95	χ^2^
Normal (<25 kg/m^2^)	11	9		
Overweight (≥25 kg/m^2^)	14	11		
Body fat percentage (*n*)			0.130	χ^2^
Normal	6	10		
Overweight	16	10		
Missing	3	0		
PSEQ (median (range))	48 (22–60)	46 (25–58)	0.44	M-U
Other comorbidity (*n*)			0.75	χ^2^
Yes	6	4		
No	19	16		
Use of protein (*n*)			1.00	χ^2^
Yes	15	12		
No	10	8		
Use of creatine (*n*)			0.64	χ^2^
Yes	13	9		
No	12	11		
Fasciotomy > 1 year ago (*n*)			0.41	χ^2^
Yes	3	0		
No	22	20		
Smoking (*n*)			0.95	χ^2^
Yes	11	9		
No	14	11		
Alcohol use (*n*)			0.24	χ^2^
Yes	16	16		
No	9	4		
Foot alignment (*n*)			0.11	χ^2^
Normal	13	15		
Abnormal	12	5		
Complaint duration (months, *n*)			0.53	χ^2^
3–6	10	4		
7–10	3	4		
11–12	2	2		
> 12	10	10		
Recurrence (*n*)			0.49	χ^2^
Yes	10	6		
No	15	14		
Physical demands (*n*)			0.57	χ^2^
Low	14	9		
High	11	10		
Missing	0	1		

*IMP* Intramuscular pressure, *BMI* Body mass index, *IQR* Inter-quartile range, *M-U* Mann-Whitney *U* test, *T* Student’s *t* - test, *PSEQ* Pain self-efficacy questionnaire, *χ* ^*2*^ Chi-Square test

Ultimately, there were 25 patients with a successful outcome after rehabilitation and 20 patients with an unsuccessful outcome. The baseline assessment and outcomes after 6 weeks of rehabilitation are presented in Table 2.

**Table 2:** Baseline assessment and outcome measurements at 6 weeks (*x* ± SD)

Item	Successful (*n* = 25)			Unsuccessful (*n* = 20)		
	Baseline	6 weeks	*P* value	Baseline	6 weeks	*P* value
Severity of leg pain on the VRS during capacity test	4.38 ± 2.22	1.22 ± 1.50	0.01	5.30 ± 2.60	4.88 ± 2.43	0.21
Severity of leg pain on the NRS at current moment	1.20 ± 1.16	0.68 ± 1.18	0.02	1.35 ± 1.98	1.20 ± 1.64	0.94
Severity of leg pain on the NRS for past week	3.76 ± 3.14	2.8 ± 2.24	0.14	3.95 ± 2.63	4.65 ± 2.54	0.39
Disability for activities on the PSFS	55.52 ± 23.72^a^	16.10 ± 12.02^a^	0.01	64.76 ± 17.66	39.61 ± 20.79	0.01

*VRS* Verbal rating scale, *NRS* Numeric rating scale, *PSFS* Patient specific functional scale

^a^represents significant difference between successful group and unsuccessful group

At baseline the successful group reported a lower VRS on the capacity test than the ‘unsuccessful’ group, however, the difference was not significant. Likewise, no significant differences between the successful and unsuccessful groups were observed at baseline for the items severity of leg pain on the NRS current moment and for past week. Only for disability in daily activities on the PSFS, the difference at baseline was significant.

At 6 weeks, the successful group experienced a non-significant reduction compared with the unsuccessful group in severity of leg pain during the capacity test. For current pain and past week pain significance was also not observed. For the disability for activities on the PSFS there were significant difference between the successful and unsuccessful groups.

In the successful group significant differences were observed for the severity of leg pain on the capacity test, the current pain and disability for activities between baseline and at 6 weeks. Only the severity of leg pain for the past week showed no significant decrease. Notably, in the unsuccessful group, only disability in daily activities, as measured by the PSFS, significantly lower at 6 weeks compared with that at baseline.

A univariate logistic regression analysis was performed to evaluate the magnitude of the association between prognostic factors and an unsuccessful outcome (Table 3). Factors exhibiting an odds ratio > 1 for an unsuccessful outcome included smoking, alcohol use, intramuscular pressure, complaint duration >6 months, and the physical demands of service. Although the results have potential clinical relevance, significance was not observed.

**Table 3:** Univariate regression analysis for CECS prognostic factors

Item	*OR* (95% CI)	*P* value
Age	0.98 (0.86–1.12)	0.76
BMI	0.98 (0.83–1.17)	0.86
Body fat	0.85 (0.71–1.01)	0.07
Self-efficacy (PSEQ)	0.97 (0.92–1.03)	0.38
Alignment of the foot	0.36 (0.10–1.3)	0.19
Intramuscular pressure	1.01 (0.97–1.05)	0.72
Other comorbidity	0.79 (0.19–3.03)	0.75
Use of protein	1.00 (0.30–3.21)	1.00
Use of creatine	0.76 (0.32–2.46)	0.64
Smoking	1.04 (0.31–3.40)	0.95
Alcohol	2.25 (0.57–8.82)	0.25
Duration of complaint	2.67 (0.69–10.36)	0.16
Demands of duty	1.41 (0.43–4.69)	0.57
Duration of military service	1.00 (0.99–1.01)	0.77

*BMI* Body mass index, *PSEQ* Pain self-efficacy questionnaire

## Discussion

This study aimed to identify prognostic factors for an unsuccessful treatment outcome in servicemen with CECS who were treated with a rehabilitation program at a military rehabilitation center. The primary outcome of the rehabilitation program for non-operative CECS demonstrated that, in the short term, a modest result could be obtained. Interpretation of these results is difficult given that the study population consists of patients who failed to be rehabilitated after monodisciplinary conservative therapy at their unit. These patients were initially referred to the special unit for lower leg pain at the Central Military Hospital to consider surgical treatment as the obvious option. However, these patients were offered an alternative option, via this study, to participate in a multidisciplinary conservative approach delivered at a military rehabilitation center.

Recently, it has been argued that the use of secondary outcome measurements for ‘current NRS’ and ‘NRS in the past week’ is questionable in this context. CECS patients experience pain that develops specifically during exercise, which is an issue that is not addressed by ‘current NRS’ and is only partially addressed by ‘NRS in the past week’ [23]. In the successful group, the decrease in pain during the functional capacity test from baseline to the 6-week follow-up was significant.

This is the first study exploring prognostic factors for CECS. In the literature, no previous studies have examined the relationship between prognostic factors and the outcomes of conservative treatment. Only one study has evaluated risk factors for CECS. Increasing age, female sex, Caucasian race, junior enlisted rank, and army service were identified as factors that significantly increased the risk of CECS. We considered adopting these risk factors as prognostic factors in our explorative study, yet the number of individuals meeting such criteria in our sample was insufficient [1].

In a ‘nonmilitary’ cohort of conservatively treated patients, outcomes were poor (success rate 41.0%) compared to a cohort undergoing fasciotomy (success rate 81.0%) [19]. However, treatment selection in this study was based on patient preference, rather than randomization. All other studies about conservative treatment have focused on massage and additional stretching techniques [15], gait retraining [20], and altering running technique from hind foot-striking to forefoot-striking are case-series [16–18]. Furthermore, it should be noted that interventions focusing on running technique are somewhat less applicable to a military study population, in which we observed marching to be the most important form of exercise in inducing CECS pain.

Military populations appear to be associated with poorer outcomes after fasciotomy for CECS than civilian cohorts with a success rate between 47.0% and 73.0% [13]. Return to duty fails for approximately 30.0%. Such results underline the differences between military and civilian populations, which presumably occur due to the physical demands of service and effects of military boots on lower limb function. Therefore, broad recommendations from surgeons despite a lack of robust evidence for the effectiveness of non-operative treatment modalities, remain understandable.

Several limitations within this study must be addressed. First, this study is limited by the relatively small sample size of 45 patients, due to the lack of eligible patients with CECS. Because of the limited cohort, a multivariate analysis and prediction model were not possible. However, the results of this study will inform calculations for sample size requirements in future studies that will include more patients undergoing conservative treatment. Second, outcome measurements were exclusively short-term in nature. Since many patients were not able to return to duty at the end of the rehabilitation program, they continued the program within their own unit. Long-term outcome measurements, such as return to duty, are required for a more rigorous assessment of conservative treatment. Furthermore, activity levels, such as aerobic fitness, marching, lifting heavy loads, and field exercises cannot be measured appropriately. These elements, which cannot be controlled for, reflect the limitations of investigating this specific patient population. The number of patients who receive surgical treatment after the rehabilitation program is also an important issue. Third, we did not repeat the IMP measurement after rehabilitation. We refrained from selecting IMP measurements as a predictive factor due to continuing broad debate regarding the relevance of IMP as an indicator for surgery and as an outcome measurement tool [7, 13]. Finally, in earlier studies, it has been argued whether the results observed in this context can be generalized to other populations [13, 32]. Indeed, the physical demands of military service, especially at the level of recruits, are exceptional. As noted above, junior enlisted personnel demonstrate the highest incidence of CECS compared to junior officers and senior enlisted personnel [1].

## Conclusions

Our study confirms modest short-term results for conservative treatment of CECS, as demonstrated in previous studies. However, we were unable to identify significant prognostic factors predicting the outcome of a rehabilitation treatment program for CECS. Since this study is predominantly limited by the relatively small sample size, a larger cohort using the same design might lead to identification of prognostic factors that are significantly related to conservative treatment outcomes. Given that obtained large samples of eligible patients is unlikely, a multicenter study design would be preferable for further research in this domain.

## Abbreviation

BMI: Body mass index
CCMO: Centrale Commissie Mensgebonden Onderzoek
CECS: Chronic exertional compartment syndrome
CI: Confidence interval
DGO: Defensie Gezondheidszorg Organisatie
FPI: Foot posture index
IMP: Intramuscular pressure
IQR: Inter-quartile range
NAF: Netherlands Armed Forces
NRS: Numeric rating scale
PSEQ: Pain self-efficacy questionnaire
PSFS: Patient specific functional scale
VRS: Verbal rating scale
